# The ProFitMap-neck - a questionnaire for measuring symptoms and functional limitations in neck pain: reliability, validity and cross-cultural adaptation of the Turkish version

**DOI:** 10.3906/sag-1912-43

**Published:** 2020-06-23

**Authors:** Hatice ÇETİN, Nezire KÖSE, Sevil BİLGİN, Haluk TEKERLEK, Esra DÜLGER, Ceyhun TÜRKMEN, Jale KARAKAYA

**Affiliations:** 1 Faculty of Physical Therapy and Rehabilitation, Hacettepe University, Ankara Turkey; 2 Department of Biostatistics, Faculty of Medicine, Hacettepe University, Ankara Turkey

**Keywords:** Neck, pain, ProFitMap-neck, Turkish, validity

## Abstract

**Background/aim:**

The Profile Fitness Mapping neck questionnaire (ProFitMap-neck) is a reliable and valid assessment instrument for measuring neck-related symptoms and functional limitations in people with neck pain, but a Turkish version of it had not been published. The purpose of this study was to investigate the adaptation, validity, and intrarater reliability of the Turkish version of the ProFitMap-neck.

**Materials and methods:**

Two hundred and thirty-five individuals with chronic neck pain were enrolled in the study. Intrarater reliability was assessed by intraclass correlation coefficient (ICC) and Cronbach’s alpha was calculated for internal consistency. For concurrent validity, ProFitMap-neck scores were compared with neck disability index (NDI) and visual analoguepain scale (VAS) scores using Pearson’s correlation coefficient analysis. The ProFitMap-neck, NDI, VAS, and short form health survey (SF-36) were administered to all participants.

**Results:**

For intrarater analysis, ICC ranged between 0.72 and 0.84. The total score was 0.83, indicating excellent reliability. The correlation of the ProFitMap-neck with NDI and VAS was 0.71 and 0.68, respectively, indicating good concurrent validity.

**Conclusion:**

The ProFitMap-neck is an evaluation instrument with sufficient validity and reliability to be used for evaluating Turkish patients with neck pain. Use of this scale can reveal how, how often, and how much these patients’ pain affects their symptoms and functional activities.

## 1. Introduction

Neck pain occurs commonly throughout the world and causes substantial disability and economic cost [1]. The prevalence of neck pain was reported to be 20.3% in 2017 and it varies between countries [2]. Pathophysiological mechanisms are lacking and risk factors are multifactorial for most cases of neck pain [3,4]. A reliable and responsive assessment of the pain of patients with neck pain is an essential prerequisite to setting realistic goals for treatment and rehabilitation, as well as for assessing the outcome of treatment interventions [5]. Therefore, a questionnaire for measuring pain, other symptoms, and functional limitations is necessary to decide on the most effective treatment method for neck pain. An important property of questionnaires is how well they mirror typical problems of the target group [6]. In addition, neck pain patients should be assessed in a biopsychosocial framework for the planning of treatment programs. According to the international classification of functioning, disability and health (ICF), questionnaires encompassing body functions, activity, and participation allow treatment programs to be composed more accurately [7].

Clinical trials showed that even if neck pain symptoms are mild and comorbidities are few, patients could have functional limitations in their daily life [8]. For this reason, pain and functional limitations are distinct domains and recommended to be measured separately for detecting differences in each domain [9]. Distinct items that focus on different domains in the same index, such as pain/symptoms and functional limitations in the neck disability index (NDI), Copenhagen neck functional disability scale, Bournemouth neck questionnaire, and Northwick Park neck pain questionnaire, may hamper detailed evaluation of treatment [10,11]. The disadvantage of having different domains in the same index is the lack of change in total score, although the domains within change separately, because the patient can improve in one domain and worsen in another. Thus, it appears advantageous to have separate indices for pain/symptoms and functional limitations, as well as a total score.

In the light of this information, questionnaires for neck pain need to include separate indices. The Profile Fitness Mapping neck questionnaire (ProFitMap-neck) meets this need. This questionnaire has the advantage of detailed assessment of symptoms and functional limitations since it consists of 2 subscales: a symptom scale, with a further subdivision in separate indices for intensity and frequency of symptoms, and a functional limitation scale [12]. There are some neck pain questionnaires that have been confirmed to be valid and reliable for Turkish patients, for example the NDI, Bournemouth neck questionnaire, and Northwick Park neck pain questionnaire [13]. However, these questionnaires have limited coverage of ICF components and categories of importance [14]. For example, while the Bournemouth neck questionnaire includes only neck pain, difficulties concentrating, and emotional engagements [15], the NDI includes symptoms from the rest of the body, such as headaches, and difficulties sleeping and concentrating [16]. On the other hand, the ProFitMap-neck includes the neck, arm, and hand; symptoms apart from pain (stiffness, tension, cracks, tiredness, weakness, lockings); symptoms from the rest of the body (fumblingness, numbness, disturbance of balance, swallowing, breathing);and mental/cognitive and emotional engagements [6]. 

The advantages of the ProFitMap-neck are that it evaluates the patients in a biopsychosocial framework comprehensively and mirrors the improvements in patients in different domains. However, there is noTurkish version. For this reason, the aim of the present study was to investigate the reliability and validity of a Turkish version of the ProFitMap-neck in neck pain patients.

## 2. Materials and methods

### 2.1. Study design

This study was conducted in City name/Country name. It was approved by the XXX University, Non-Interventional Clinical Trials Ethics Committee (Approval no: GO 16/235) and registered in the Clinical Trials database (NCT03415737). Written permission was obtained from questionnaire developers for the Turkish version of the ProFitMap-neck, and the translation and cultural adaptation were carried out according to the procedure established by Beaton et al.[17]. Details of each step are explained in the following part. Two hundred andforty native Turkish speaking individuals participated in the study. The sample size of this study was chosen as 5 times the number of items used in the scale [18]. The study was completed performing the reliability and validity analysis. The flowchart of the study was shown in Figure. 

**Figure:**
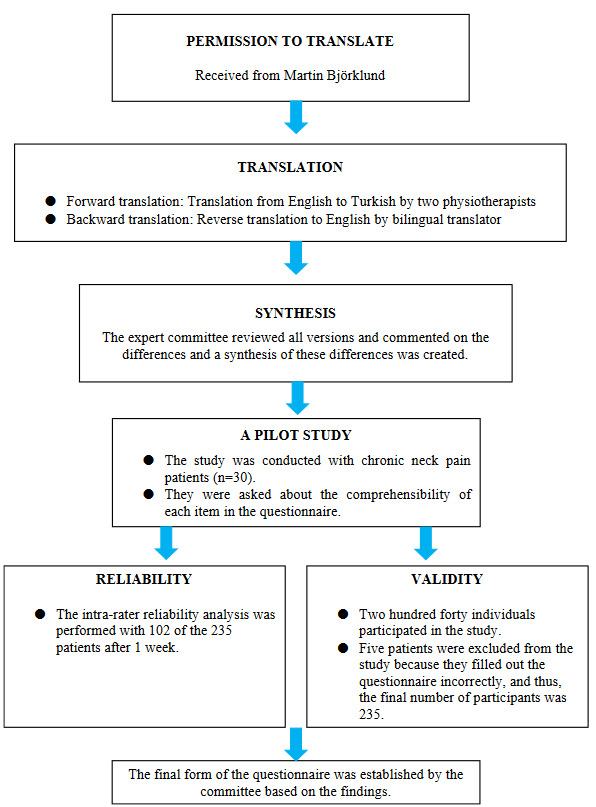
The flowchart of the study.

### 2.2. Translationand cultural adaptation 

Details of each step are explained below: 

**Step 1: Liaison with the ProFitMap-neck’s developers **

Contact was established via mail with Dr. Björklund at the Department of Occupational and Public Health Sciences, University of Gävle, Sweden, the first author of the original validation study of the ProFitMap-neck [12]*. *The purpose was to determine whether there were any attempts in progress to develop a Turkish version of the instrument. 

**Step 2: Translation (English to Turkish) **

The original English form of the questionnaire was translated into Turkish by 2 native Turkish speakers with good command of English. One of them was a physiotherapist and was aware of the study, while the other was an English linguistic scientist unaware of the concepts. 

**Step 3: Back-translation (Turkish to English)**

The 2 versions of the Turkish translation were combined into a single translation by the 2 translators. This combined Turkish version of the questionnaire was then translated back into English by 2 bilingual translators (back-translation). The bilingual translators were unaware of the study.

**Step 4: Synthesis**

The expert committee consisted of 2 physiotherapists, 2 bilingual translators, and a specialist in public health science. The committee reviewed all versions. The content of the original and reverse-translated English versions was compared and differences were noted. The reviewers commented on the differences and a synthesis of these differences was created. 

**Step 5: Consensus building**

Following the evaluation of the resultant translations for English–Turkish language and cultural adaptation by the expert committee, the prefinal form of the questionnaire was created. 

**Step 6: Pilot testing**

The comprehensiveness of the questionnaire was evaluated in a pilot group of 30 people (15 patients/15 healthy individuals) and they were asked about the comprehensibility of each item in the questionnaire (face validity). 

**Step 7: Development of the final version **

After the pilot group completed the questionnaire, the final form of the questionnaire was established by the committee based on the findings.

### 2.3. Participants

Individuals were recruited from the Department of XXX at the XXX University and from the campus of the university, via advertisement. Individuals 18 to 65 years of age who had a primary problem of neck pain that had persisted for 12 weeks or more, who had good verbal communication, and who had the ability to read and write in Turkish were included. The exclusion criteria were having vestibular, neurological, musculoskeletal, and cardiovascular disease; having a history of cervical surgery; and being pregnant.Informed consent was obtained from all individual participants included in the study.

### 2.4. Instruments 

#### 2.4.1. Profile Fitness Mapping (ProFitMap) neck questionnaire

The ProFitMap-n was designed by an expert group of health professionals at the Alfta Rehab Center, a rehabilitation clinic in mid-Sweden, in 1992–1994 for measuring symptoms and functional limitations in people with the most prevalent categories of neck pain. This questionnaire consists of 2 subscales: a symptom scale (27 items) and a functional limitation scale (20 items). The symptom scale also consists of 2 indices of separate aspects of symptomatology, the intensity and the frequency of the symptoms, and the functional limitation scale yields 1 function index. Frequency (f) is the answer to howoftenthe symptom is felt (6-point scale from 1 = never/very seldom, to 6 = very often/always). Intensity (i) is the answer to how much the symptom is felt (6-point scale from 7 = nothing/none at all, to 12 = almost unbearable/unbearable, all/maximally). The answers for the functional limitation scale range from 1 = very good, no problem, very satisfying, very likely, to 6 = very bad, very difficult/impossible, very dissatisfying, very unlikely. The result of each index is expressed as the percentage of the maximum score, where 100% is the best possible result. Thus, 3 index percentages and a total percentage are obtained from this questionnaire. See Björklund et al. for the questionnaire form and method of score calculation of the ProFitMap-neck [12]. 

#### 2.4.2. Neck disability index (NDI)

The NDI is the most commonly used outcome measure for neck pain and it contains 10 subsections consisting of severity of pain, personal care, lifting, reading, headache, concentration, work, driving, sleeping, and leisure activities. The questions are measured on a 6-point scale from 0 (no pain and functional limitation) to 5 (worst pain and maximal limitation). The numeric response for each item is summed for a score varying from 0 (no disability) to 50 (totally disabled) [16,19]. 

#### 2.4.3. Visual analogue scale (VAS)

A VAS is a vertical line, 100 mm in length, with the bottom of the line indicating “no pain” and top of the line the worst pain; the possible score lies between 0 and 10. The VAS was used to assess the subjects’ pain [20]. 

#### 2.4.4. Short form health survey (SF-36)

This survey instrument is designed for use in clinical practice and research, health policy investigations, and general population examinations [21]. The SF-36 includes one multiitem scale that assesses 8 health concepts with 36 items: physical functioning (PF), general health (GH), emotional role limitations (ERL), vitality (V), physical role limitations (PRL), social functioning (SF), and bodily pain (BP). Each question’s score was coded, summed, and transmuted to a scale of 0 (worst possible health state measured by the questionnaire) to 100 (best possible health state) [22]. 

### 2.5. Statistical analysis

#### 2.5.1. Reliability

Internal consistency: 

Cronbach’s alpha was utilized for the internal consistency analysis. A cronbach’s alpha value of 0.80 or higher is considered excellent [23].

Intrarater Reliability: 

For the intrarater reliability, the questionnaire was completed twice. The period between measurements was 7 days. Intrarater reliability was determined by using the intraclass correlation coefficient (ICC). ICCs can vary from 0.00 to 1.00, with values of 0.60 to 0.80 regarded as evidence of good reliability and those above 0.80 indicating excellent reliability [23].

#### 2.5.2. Validity

The concurrent validity was examined by comparing the total scores of the ProFitMap-neck with those of the NDI, VAS, and SF-36. Concurrent validity coefficients were regarded as follows: r ≥ 0.81–1.0 as excellent, 0.61–0.80 very good, 0.41–0.60 good, 0.21–0.40 fair, and 0–0.20 poor [23]. The relation was evaluated with Pearson’s correlation coefficient.

## 3. Results

Two hundred and forty people, aged between 18 and 65, participated in the study. Five patients were excluded because they filled out the questionnaire incorrectly, and thus the final number of the participants was 235. The mean age of the included subjects was 33.49 ± 15.17 years, and 164 (69.8%) were female and 91 (30.2%) were male. Detailed demographic data are listed in Table 1.The intrarater analysis was performed with 102 of the 235 patients after 1 week, and they received no treatment for 7 days.

**Table 1:** Baseline participant demographics (n = 235).

Variable	Mean ± SD	n (%)
Age (years)	33.49 ± 15.17	
Sex Female Male		164 (69.8) 91 (30.2)
Height (cm)	167.24 ± 8.56	
Weight	69.06 ± 14.96	
BMI	24.63 ± 4.8	
Education Elementary-mid school High school Graduate school		37 (15.8) 59 (25.1) 139 (59.1)
VAS (0–10)	4.61 ± 1.92	
NDI (0–50)	13.02 ± 7.21	
SF-36 PF (0–100) a GH (0–100) a ERL (0–100) a V (0–100) a PRL (0–100) a SF (0–100) a BP (0–100) a	72.10 ± 18.23 55.76 ± 37.11 56.51 ± 32.83 49.70 ± 16.7062.66 ± 16.0667.37 ± 18.8361.70 ± 17.72	
ProFitMap-neck Symptom frequency index (0–100) aSymptom intensity index (0–100) aFunction index (0–100) aTotal score (0–100) a	70.62 ± 13.9074.43 ± 12.8467.71 ± 17.4471.33 ± 12.81	

a0 = Worst score and 100 = Best score. BMI: Body mass index; VAS: Visual analog scale; NDI: Neck disability index; ProFitMap-neck: Profile fitness mapping neck questionnaire; SF-36: Short form health survey; PF: Physical functioning; GH: General health; ERL: Emotional role limitations; V: Vitality; PRL: Physical role limitations; SF: Social functioning; BP: Bodily pain.

### 3.1. Translation and cross-cultural adaptation 

During the translation and back-translation, the main changes made to the symptom scale were question sentences were added to items 25 and 26 in order to make them easier to understand. For the functional limitation scale, the word “weight (ağırlık, in Turkish)” was added to items 6 and 7 to make “carry weight (ağırlık taşımak, in Turkish)” and “lift weight (ağırlık kaldırmak, in Turkish)”. Moreover, “throw” was changed to “throw stuff”. The word “sweater” was culturally adapted to “T-shirt/sweater (tişört/kazak, in Turkish)” because of the changeable weather conditions in Turkey (Appendix).

### 3.2. Reliability

#### 3.2.1. Internal consistency

For the reliability analysis, Cronbach’s alpha values of the ProFitMap-neck indices were recorded as follows: for symptom frequency index 0.894, for symptom intensity index 0.899, for functional index 0.943, and for total score 0.959, indicating that the questionnaire has high internal consistency (Table 2). 

**Table 2:** The internal consistency of the ProFitMap-neck indices.

	Symptom Frequency index	Symptom Intensity index	Function index	Total score
Cronbach’s alpha	0.894	0.899	0.943	0.959

ProFitMap-neck, the profile fitness mapping neck questionnaire.

#### 3.2.2. Intrarater reliability

The ICC values ranged from 0.725 to 0.841 (Table 3). The ICC values of the ProFitMap-neck were recorded as follows: for symptom frequency index 0.841, for symptom intensity index 0.725, for functional index 0.797, and for total score 0.830 (Table 3). According to the ICC values, the ProFitMap-neck test-retest (intrarater) results were excellent. 

**Table 3:** The intraclass correlation (ICC) coefficients values of ProFitMap-neck.

ProFitMap-neck	ICC (95% confidence interval) Lower-upper bound
Symptom frequency index	0.841 (0.773–0.889)
Symptom intensity index	0.725 (0.619–0.806)
Function index	0.797 (0.714–0.858)
Total score	0.830 (0.758–0.882)

### 3.3. Validity

The correlation coefficients between the ProFitMap-neck indices and the criterion questionnaires are presented in Table 4. For validity, the correlation of total scores of the ProFitMap-neck with the NDI was r: 0.710 and with the VAS was r: 0.68. The correlations between the symptom frequency index, symptom intensity index, and functional index, which were the subparameters of the ProFitMap-neck indices, and the NDI were r: 0.682, r: 0.612, and r: 0.654, respectively. Based on these results, the ProFitMap-neck had a very good correlation with the NDI. The correlations between the total scores of the ProFitMap-neck and the SF-36 indices varied between poor and good (0.18–0.52) (Table 4). 

**Table 4:** The bivariate correlations between the ProFitMap-neck index scoresa and the scores of the criterion questionnaires.

	Symptom frequency index r (P-value)	Symptom intensity index r (P-value)	Function index r (P-value)	Total scorer (P-value)
VAS	–0.500**	–0.499**	–0.518 **	–0.684**
NDI	–0.612 **	–0.682 **	–0.654 **	–0.710 **
SF-36 PF GH ERL V PRL SF BP	0.398** 0.331** 0.193** 0.292** 0.303** 0.359** 0.481**	0.404** 0.275* 0.138** 0.244** 0.287** 0.294** 0.417**	0.532** 0.374** 0.159* 0.310** 0.201** 0.367** 0.421**	0.522** 0.357** 0.188** 0.317** 0.287** 0.378** 0.481**

aPearson rank correlation. *P < 0.05 , **P < 0.001. ProFitMap-neck: Profile fitness mapping neck questionnaire; SF-36: Short form health survey; PF: Physical functioning; GH: General health; ERL: Emotional role limitations; V: Vitality; PRL: Physical role limitations; SF: Social functioning; BP: Bodily pain; NDI: Neck disability index.

## 4. Discussion

This study demonstrated the reliability and validity of the Turkish version of theProFitMap-neck. The analyses support the reliability and validity of the instrument for Turkish neck pain patients. 

The present study shows that the Turkish version of the ProFitMap-neck has good internal consistency. This was compatible with the internal consistency level usually found and deemed appropriate for other measures (>0.7) (symptom frequency 0.894, symptom intensity 0.899, function index 0.943, and total score 0.95).

For intrarater reliability, ICCs were only reported in the original and Portuguese versions [12], [24]. While 210 patients were enrolled in the original version, 180 female patients with chronic neck pain participated in the Brazilian (Br) Portuguese version. Strong reliability was identified by high ICCs (ICC > 0.75). In the present study, we found that the total ICC value was 0.830 at 1week for the intrarater reliability intervals (ICC values above 0.80 showed excellent reliability). The ICC value for each index varied between 0.72 and 0.84. As a result, it appears that the Turkish version of the ProFitMap-neck is highly stable over time. The ICC values ranged from 0.81 to 1 in the Br-Portuguese version of ProFitMap-neck [24]. The researchers indicated that the Br-ProFitMap-neck had high levels of reliability for total score and indices. They also explained that the possible reason for the high reliability in their study was the short time interval between test and retest (at least 5 h), which allows for close control of the clinical stability of the patients. In addition, they indicated that future studies should test the Br-ProFitMap-neck by using longer test–retest intervals (between 1 and 2 weeks).We had a 1-week time interval between test and retest and excellent reliability in our study. Therefore, our study evaluated fluctuations in the functioning/disability and symptoms that are important for clinical trials as stated in the validity study of the Br-ProFitMap-neck. 

For validity, the present study assessed the correlation between the ProFitMap-neck and the NDI, VAS, and SF-36. We demonstrated the Pearson’s correlation coefficient value of the ProFitMap-neck with the NDI was 0.71. Furthermore, all indices of the ProFitMap-neck showed good correlation with the NDI (r for symptomintensity index: 0.61; r for symptomfrequency index: 0.68; r for function scale: 0.65; and r for total score: 0.71). The Pearson’s correlation coefficient values showed that the correlation of the Turkish version of ProFitMap-neck with the NDI and VAS was high.

In the Br-Portuguese version of the ProFitMap-neck, the correlation values between the domains of the Br-ProFitMap-neck and NDI varied from 0.56 to 0.71 [24]. The results of our study were similar to those for the Br-Portuguese version of the ProFitMap-neck. Furthermore, our results are consistent with those of the study of the original version [12].

The correlation value between the ProFitMap-neck and VAS was 0.68 in our study.When we analyzed the other Turkish version scales, the correlations between total score of the mean VAS and Copenhagen neck functional disability scale, neck pain and disability scale, Northwick Park pain questionnaire were r = 0.72, r = 0.83, r = 0.78, respectively [13]. Aslan et al. showed that the relation value between NDI and VAS was 0.62 in their study [15]. These results are similar to our current study. The correlations between the SF-36 and ProFitMap-neck, we found fair and good correlations. In particular, the correlation value between the ProFitMap-neck and the emotional role limitation indices of SF-36 was 0.18, which indicates poor correlation. This correlation value in the original study was 0.38 [12]. The perceived disability of patients included in our study was 13.02 (indicates minimal disability), whereas in the original study it was 14.2 (indicates mild disability). We think that a minimal level of disability may not cause emotional role limitations. The correlation values between the ProFitMap-neck and the other subscales of SF-36 were also similar to those of the original study. 

Neck pain occurs commonly throughout the world and causes substantial disability and economic cost. The pain and disability associated with neck pain have a large impact on individuals and their families, communities, healthcare systems, and businesses. Economic consequences include the cost of healthcare, reduced work productivity, work absenteeism, and insurance [25,26]. Therefore, choosing the most convenient assessment tool can make the planning of treatment programs for patients with neck pain easier. The separate scores for specific domains, preferably combined with an overall judgment score, as in the ProFitMap-neck, may be considered advantageous not only in clinical practice but also in research. Therefore, the current study has importance for biopsychosocial examinations of Turkish populations with neck pain.

In conclusion, the ProFitMap-neck is an evaluation instrument with sufficient validity and reliability to be used for evaluating Turkish neck pain patients. Use of this scale can reveal how, how often, and how much patients’ pain affects their symptoms and functional activities, which will play a key role in managing patients with neck pain.

## Acknowledgments/Disclaimers

The authors thank Dr. Martin Björklund (Faculty of Health and Occupational Studies, University of Gävle, Gävle, Sweden) for his permission to translate the ProFitMap-neck into Turkish, and the members of the committee (Jern Hamberg, Marina Heiden, Margareta Barnekow-Bergkvist) for their cooperation. 

The study was approved by Ethics Committee (Approval no: GO 16/235) and registered in the Clinical Trials database (NCT03415737). No support has been received for this study.

## Conflict of interest

The authors report no conflicts of interest. 

## Informed consent

Written informed consent was obtained from all study participants.
